# Risk factors for multiple sclerosis in the context of Epstein-Barr virus infection

**DOI:** 10.3389/fimmu.2023.1212676

**Published:** 2023-07-24

**Authors:** Anna Karin Hedström

**Affiliations:** Department of Clinical Neuroscience, Karolinska Institute, Stockholm, Sweden

**Keywords:** multiple sclerosis, Epstein-Barr virus, infectious mononucleosis, human leukocyte antigen, vitamin D, sun exposure, smoking, obesity

## Abstract

Compelling evidence indicates that Epstein Barr virus (EBV) infection is a prerequisite for multiple sclerosis (MS). The disease may arise from a complex interplay between latent EBV infection, genetic predisposition, and various environmental and lifestyle factors that negatively affect immune control of the infection. Evidence of gene-environment interactions and epigenetic modifications triggered by environmental factors in genetically susceptible individuals supports this view. This review gives a short introduction to EBV and host immunity and discusses evidence indicating EBV as a prerequisite for MS. The role of genetic and environmental risk factors, and their interactions, in MS pathogenesis is reviewed and put in the context of EBV infection. Finally, possible preventive measures are discussed based on the findings presented.

## Introduction

Multiple sclerosis (MS) is an inflammatory and neurodegenerative disease of the central nervous system (CNS), with both genetic and environmental factors contributing to its development. While significant progress has been made in identifying genetic risk factors for MS, these genetic variants explain only a proportion of the heritability, which is estimated to be around 50% (1). This missing heritability phenomenon may partly be explained by the influence of multiple genetic variants on the risk of the disease, each with a small or modest effect, making it challenging to identify them. Epigenetic modifications, which can influence gene expression without altering the DNA sequence, may also play a role (2, 3). Furthermore, environmental factors implicated in the disease may interact with genetic and other environmental exposures or lifestyle habits to increase the risk of the disease (4). Thus, the complex interplay between multiple genetic variants, epigenetic modifications, and environmental factors may contribute to explain the missing heritability in MS. Compelling evidence suggests that infection with Epstein-Barr virus (EBV), a gamma-herpes virus that infects more than 90% of the population, is a prerequisite for developing MS. Several aspects of EBV infection, such as high EBV-specific antibodies and infectious mononucleosis (IM), have consistently been associated with increased MS risk (5, 6). Nearly all patients with MS are EBV-seropositive, whereas the risk of the disease in seronegative individuals is extremely low (7). In a seronegative group of young adults in the US military, the risk of MS increased by 32-fold following infection with EBV, strongly indicating a causal link (8). However, since most EBV seropositive individuals will never develop MS, inadequate control of the virus may be another prerequisite for the disease to occur. Several environmental and lifestyle factors have been implicated in MS such as vitamin D, smoking, and obesity (4). According to the sufficient cause model by Rothman, a disease occurs when multiple risk factors act together and reach a threshold level of causality, which then trigger the disease process (9). The model is a useful framework for studying complex diseases as it emphasizes the importance of multiple risk factors interacting to cause disease and can help to identify high-risk populations and guide the development of targeted interventions and preventive strategies. This review gives a short introduction to EBV and host immunity and discusses evidence indicating that EBV is a prerequisite for MS. The role of genetic and environmental risk factors, and their interactions, in the development of MS are reviewed and put in the context of EBV infection. Finally, possible preventive measures are discussed based on the findings presented.

## Epstein-Barr virus – background

EBV lytically infects epithelial cells and B lymphocytes. The virus has a double-stranded DNA genome enclosed by an icosahedral capsid, a tegument layer, and an outer lipid envelope containing the viral attachment and entry proteins. Infection of B lymphocytes involves the attachment of the viral envelope glycoprotein gp350/220 to the complement receptor CD21 on B lymphocytes, followed by the interaction of viral gp42 with MCH class II molecules (10). This interaction leads to fusion of the viral envelope with the cellular membrane, allowing the viral capsid to enter the cell. Once inside the cell, the nucleocapsid is transported to the nucleus where viral DNA is released and begins to replicate. Following primary infection, EBV persists latently throughout life, mainly in memory B lymphocytes (11). During latency, the virus enters a state of limited gene expression which enables it to evade host immune surveillance (12). A balance is typically established between viral persistence and immune control, although reactivation of the virus occurs periodically, usually without symptoms (13).

## Immune response to EBV

Primary EBV infection is usually asymptomatic in childhood. Subsets of natural killer (NK) cells become activated within one or two days as a first-line defense, playing a crucial role in limiting viral spread. EBV-specific CD8+ T cells are responsible for eliminating proliferating and lytically infected B cells while EBV-specific antibodies neutralize viral infectivity by binding to free virus (14, 15). During latency, the number of infected memory B cells varies between individuals but remains rather stable throughout life, reflecting a dynamic equilibrium between viral persistence and EBV-specific immune responses. Healthy EBV carriers typically show IgG-antibody titers against the viral capsid (VCA) and Epstein-Barr virus nuclear antigens (EBNAs) (16).

When primary EBV infection is delayed beyond childhood, it often manifests as acute IM, indicative of a reduced capacity of the CD8+ T cell response to rapidly control the infection (17). The symptoms of IM are caused by an exaggerated cellular immune response to EBV, which may be influenced by host genetics (18, 19) and an age-related impaired CD8+ T cell function (20–22). Furthermore, early NK cell-mediated control over primary EBV infection decreases during the first decade of life which may contribute to the development of an exaggerated CD8+ T cell response when the infection is acquired at an older age (23, 24).

In IM, the number of latently infected memory B cells can rise to half of the peripheral memory B cell compartment. Latent infection is only achieved after a massive expansion of CD8+ T cells that target EBV proteins from the early stages of the lytic cycle. The CD8+ T cell expansion coincides with the onset of symptoms. In addition, cytotoxic CD4+ T cell populations are also amplified and contribute to the symptoms of acute IM by massive cytokine production (17). Although the total number of CD8+ T cells is not significantly raised in children with asymptomatic primary EBV infection, EBV-specific CD8+ T cells are highly activated and can control the infection without causing excessive expansion (14, 15), confirming that cytotoxic lymphocytes are the cornerstone of EBV-specific immune control, and that IM is an immunopathologic condition.

## Epstein-Barr virus as a necessary factor in MS

EBV has a profound influence on the immune system, and recent studies have suggested a causal role of EBV in MS. There is consistent evidence that EBV infection precedes MS onset. EBV-specific antibodies are present in nearly all patients with MS whereas the risk of MS in seronegative individuals is extremely small (7). In a seronegative group of young adults in the US military, the risk of MS increased 32-fold after infection with EBV. The study also demonstrated that serum levels of neurofilament light chain, a biomarker that reflects neuroaxonal damage, increased shortly after seroconversion (8). These findings indicate that EBV is a necessary, but not sufficient, factor in MS development.

Presence of EBV in latency has been found to be considerably more prevalent in MS brain tissue than in non-MS brain, and lytic EBV infection has been detected in chronic MS lesions (25, 26) and in meningeal B cell follicles (27, 28). Apart from infected B lymphocytes, EBV has also been detected in astrocytes and microglia in some MS cases with widespread presence of EBV in brain tissue (26). However, other studies have been unable to detect EBV in MS brain tissue and therefore, the issue of the presence of EBV in MS brain tissue remains unresolved (29–31).

Generally, patients with MS have higher levels of EBV-specific antibodies than control populations (32), which suggests that the control of latent EBV infection may be compromised in these individuals. Several studies have observed a significant increase in EBNA-1 antibody titers many years prior to the clinical onset of MS (33–35). Moreover, the risk of MS increases with increasing EBNA-1 antibody levels (36). Under normal circumstances, the immune system effectively controls EBV infection, leading to a state of viral latency where the virus remains dormant in B cells. However, an increase in the levels of EBNA-1 antibodies implies that the overall immune response may be insufficient to effectively control the virus, resulting in persistent or reactivated infection. The presence of disease-associated oligoclonal Ig-bands in the cerebrospinal fluid (CSF) is a well-established characteristic of MS, indicative of local antibody production within the CNS. In a subset of patients, EBV-specific antibody synthesis occurs within the CNS. However, it is generally observed that the frequency of EBV-specific antibodies in the CSF is lower compared to other viral infections in MS (37), which may be attributed to differences in localization of antibody production, viral tropism, or temporal dynamics of antibody response. EBV-specific CD8+ T cells present in the CNS of MS patients provide further evidence that EBV-infected cells are present in the brain (38, 39). Furthermore, a higher frequency of CD4+ T cells primarily targeting EBNA has been observed in MS patients (40, 41), where a subset of the T cells cross-reacted with CNS-antigens (41). In this context, it is conceivable that the elevated levels of EBNA-1 antibodies reflect a breakdown in immune tolerance, where the immune system fails to distinguish between viral and CNS-antigens. The presence of high levels of EBNA-1 antibodies in MS patients could thus be attributed to various factors, including the complex interplay between EBV infection, immune dysregulation, and potential cross-reactivity with CNS antigens. Further research is needed to elucidate the precise mechanisms underlying these observations and their implications in the pathogenesis of MS.

Several hypotheses have been put forward to explain the role of EBV in MS (42, 43), but the pathogenic mechanisms remain uncertain. During primary EBV infection, there is a high frequency of EBV-infected B lymphocytes. An impaired immune response to the virus may increase the viral load whereby circulating EBV-infected B cells may enter the CNS and initiate proliferation. Failure to eradicate the infection could lead to viral antigens stimulating an anti-EBV immune response with maintenance of local inflammation, intrathecal immunoglobulin synthesis and oligoclonal bands. If EBV-specific CD8+ and CD4+ T cells migrate into the CNS and become reactivated by EBV-infected B lymphocytes, the resulting immune response would lead to bystander damage and the release of CNS-antigens, which could subsequently result in a CNS-directed immune response. Alternatively, EBV-transformed B lymphocytes could activate EBV-specific T cells cross-reactive with CNS antigens. However, the exact mechanisms by which EBV increases the risk of MS are yet to be determined.

## Infectious mononucleosis

The risk of MS is increased more than two-fold following IM (5, 6), suggestive of an inadequate cytotoxic CD8+ T cell control of the infection. Several studies have observed impaired EBV-specific CD8 T cell responses in patients with MS (44, 45). Furthermore, CD8+ T cells specific for lytic EBV antigens increase during the active phase of MS whereas CD8+ T cells against latent antigens dominate during inactive disease (46). During primary EBV infection, there is a high frequency of EBV-infected B lymphocytes. An impaired immune response to the virus may, apart from increasing the risk of IM, increase the viral load whereby circulating EBV-infected B cells may enter the CNS and initiate proliferation.

Past IM does not correlate with EBNA-1 antibody levels (36, 47) and both aspects of EBV infection appear to be separate risk factors for MS. A synergistic effect between past IM and high EBNA-1 antibody levels has been observed, which indicates that they are involved in at least one common biological pathway to disease (36). Both past IM and high EBNA-1 antibody levels may thus reflect a deficient control of the EBV infection, for which cell-mediated immune responses play a pivotal role. Since most seropositive individuals will never develop MS, inadequate control of the virus may thus be another prerequisite for developing the disease.

## Risk factors for MS onset in the context of EBV

The high prevalence of EBV seropositivity in the general population and the low incidence of MS indicates that a potential pathogenic effect of the virus is influenced by other risk factors for the disease. According to the sufficient cause model by Rothman (9), the development of a disease is the result of a sufficient cause which is a set of conditions or events that act together to produce the disease. A necessary cause is a factor that must be present for the disease to occur, whereas component causes are other factors that contribute to the development of disease but are not sufficient on their own. All component causes in a sufficient cause are required to be either present or have taken place before disease onset. In the case of MS, EBV appears to be a necessary component cause. In every sufficient cause that leads to MS, EBV would thus be required. All component causes in the same sufficient cause act in concert to produce the disease. If EBV is a necessary component cause for MS to occur, it would thus be expected to interact with every other risk factor for the disease (sufficient cause interaction). The sufficient cause model recognizes that multiple pathways can lead to the same disease, and that different combinations of factors can lead to the same outcome (48). The model can be used to guide research and intervention efforts, as it emphasizes the importance of identifying and addressing all factors that contribute to the development of disease.

Interaction between two exposures is present when the effect of one exposure on the outcome depends on the other exposure. Statistical interaction evaluates whether the combined effect of the exposures deviates from either additivity or multiplicativity, depending on the scale of the model (48). Although it is not possible to draw conclusions regarding biologic mechanisms through statistical analysis of observational data, interaction on the additive scale can be used to test for sufficient cause interactions in epidemiologic research when the outcome is rare. Additive interaction (departure from additivity) means that the combined effect of two exposures differs from the sum of the risks conferred by each exposure and can be assessed by using different measures such as the synergy index, the attributable proportion due to interaction, and the relative excess risk due to interaction. The additive scale is preferable from a public health perspective since it implies that some subgroups would obtain a greater absolute risk reduction from preventive measures. Studies investigating additive interactions between risk factors in MS etiology are thus important for the development of effective preventive measures but may also shed light on disease pathogenesis. In the following, risk factors for MS and their interactions will be discussed and put in the context of EBV infection.

## Viral infections apart from EBV

In addition to EBV, several other viruses have been implicated in MS, such as human endogenous retroviruses (HERVs), human herpesvirus 6 (HHV-6) and cytomegalovirus (CMV), which all have the capacity of establishing lifelong latent infections (49). The underlying mechanisms are poorly understood. Exposure to prior antigens could result in heterologous immunity in which the immune response to a prior infection provides some degree of protection or enhances response against a different but related infection (50). The specific mechanisms by which prior infections could affect the immune response to EBV are likely complex and multifactorial and may vary depending on factors such as the timing and severity of infections as well as on the host immune status.

HHV-6, associated with increased risk of MS (49), is capable of infecting glia and neurons and is one of the most common viruses detected in the adult brain (51, 52). Regarding risk of MS, an additive interaction has been observed between high antibody levels against EBV and HHV-6A (53). Some studies have reported that HHV-6 can infect and activate EBV-infected B cells, leading to increased viral replication and persistence (54–56). Compared with controls, increased expression of both HHV-6 and HERVs have been found in MS plaques (57–59), and cumulative roles for EBV, HHV6, and HERVs in MS pathogenesis has been suggested (60).

In seroepidemiological studies, CMV has been inversely associated with MS (61, 62). CMV infection may result in changes in the CD8+ T cell repertoire with an accumulation of oligoclonal differentiated T cells with limited proliferative capacity (63), which could potentially limit the immune response to EBV. One study found a reduced proportion of memory B cells, with reduced inflammatory profile, in MS patients with previous CMV infection, compared to those without CMV infection (64). However, potential mechanisms by which viral infections contribute to MS are hypothetical and more research is necessary.

## Genetics

The genetic contribution to MS is complex, with hundreds of common genetic variants, identified by genome-wide association studies, contributing to MS heritability (1). The importance of human leukocyte antigen (HLA) class I and II alleles in MS is well established. The strongest genetic factor is the DRB1*15:01 allele in the class II region which increases the risk of MS approximately 3-fold, whereas the HLA class I allele A*02:01 confers a protective effect (65). The class II molecules present peptide ligands to CD4+ lymphocytes, which are thought to play a crucial role in the pathogenesis of MS. In addition to HLA genes, more than 200 non-HLA risk alleles have been identified as implicated in disease susceptibility (1). Most of these are associated with innate or adaptive immune functions or brain-resident immune cells. While individual genetic variants have only small or modest effects on MS risk, recent studies have shown that combining multiple genetic variants into a polygenic risk score can improve the accuracy of predicting the risk of disease (66).

Considering the role of HLA class II for the infection of B lymphocytes, variations in HLA may account for the differences in EBV susceptibility (67). EBV serostatus has been linked to the ability of EBV to bind to B lymphocytes. Since CD21 on B cells is highly conserved, the binding capacity of EBV to B lymphocytes is likely a result of variation in HLA.

Once established, both class I and II-restricted antigen presentation are involved in the control of the EBV infection. Genetic differences in the HLA class I locus have been associated with both the development and outcome of primary EBV infection as well as with viral persistence (19, 68). The frequency of A*02:01 has been shown to be lower among MS patients with past IM compared to both cases without IM history and healthy controls (36). The MS protective allele A*02:01 can present several EBV-derived peptides and mediates an effective immune response against EBV antigens (69, 70), although both B and T cells carrying this allele display a reduced response to type I IFN stimulation (71).

Carriers of DRB1*15:01 have higher EBNA-1 antibody levels compared to DRB1*15:01 negative individuals, both among MS patients and healthy controls (36, 68), indicating a defective immune response to EBV. Non-HLA loci influencing both EBNA-1 antibody levels and risk of MS have also been identified (72, 73). The class II molecules, involved in antigen-presentation to CD4+T cells by antigen presenting cells, are essential in shaping the TCR repertoire. The DRB1*15:01 allele has been associated with a narrower TCR repertoire with T cells of high affinity for specific peptides (74). Epigenetic mechanisms may also be involved, as it has been suggested that DNA methylation mediates the effect of DRB1 expression and could lead to a changed TCR repertoire more prone to CNS-reactive responses (75). In a humanized mouse model, HLA-DRB1*15-restricted CD4+ T clones were less efficient in recognizing EBV-transformed B cell lines, resulting in increased expansion of CD8+ T cells and higher viral load. The CD4+ T cell clones also demonstrated cross-reactivity with CNS-antigens (76). Furthermore, it has been suggested that the DRB1*15:01 haplotype may promote the activation of brain homing autoreactive CD4+ T cells by memory B cells (77). In MS, EBV-specific CD4+ T cells among DRB1*15:01 carriers also show higher reactivity against several CNS-antigens (78).

The findings presented may contribute to explain the interaction between HLA alleles, encoding molecules regulating T cell adaptive immunity, and measures of EBV infection, in the development of MS (79–85). Both past IM and high EBNA1 antibody levels act synergistically with the main MS risk HLA genes, presence of DRB1*15:01 and absence of A*02:01 (85). Carriers of DRB1*15:01 without the protective effect of A*02:01 and with high EBNA-1 antibody levels following IM run an almost 30-fold increased risk of MS, compared to those who have none of these risk factors. **Table 1** provides a compilation of studies examining the additive interaction between EBV and HLA-DRB1*15:01 (and absence of HLA-A*02:01) in relation to the development of MS. Studies that investigated the individual effects of each risk factor as well as their combined effects were included. The presented results are directly extracted from the original works referenced.

**Table 1:** Compilation of studies examining the additive interaction between Epstein-Barr virus and HLA-DRB1*15:01 (and absence of HLA-A*02:01) in relation to the development of MS.

Risk factors	OR for each risk factor in the absence of the other/s	Combined OR	Additive interaction	Reference
Anti-EBNA seropositive HLA-DRB1*15:01 positive	2.3 + 1.5	8.5	AP 0.7 (0.4-1.0)	78
High anti-EBNA-1 IgG HLA-DRB1*15:01 positive	2.0 + 3.0	5.8	SI 1.6, p=0.4	77
High anti-EBNA HLA-DRB1-15:01 positive	4.6 + 3.3	9.7	Not calculated	82
Anti-EBNA>median HLA-DRB1*15:01 heterozygote*	2.6 + 3.2	10.0	AP 0.5 (0.4-0.6)	83
Anti-EBNA>median HLA-DRB1*15:01 homozygote*	2.6 + 7.7	19.5	AP 0.5 (0.4-0.7)	83
Anti-EBNA>median HLA-DRB1*15:01 positive HLA-A*02:01 negative	2.3 + 3.4 + 1.6	16.8	Total RERI 11.6 (9.1-14.0)	33
Anti-EBNA>median HLA-DRB1*15:01 heterozygote* HLA-A*02:01 negative	2.2 + 3.2 + 1.4	14.9	Not calculated	83
Anti-EBNA>median HLA-DRB1*15:01 homozygote* HLA-A*02:01 negative	2.2 + 8.5 + 1.4	28.7	Not calculated	83
Anti-EBNA>median Past IM HLA-DRB1*15:01 positive HLA-A*02:01 negative	2.2 + 3.3 + 1.5 + 1.3	27.1	Not calculated	33
Past IM HLA-DRB1*15:01 positive	1.6 + 3.5	7.3	AP 0.45 (0.2-0.7) RERI 3.3 (0.5-6.1) SI 2.1 (1.6-2.6)	79
Past IM HLA-DRB1*15:01 positive	1.7 + 2.4	7.0	Not calculated	80
Past IM HLA-DRB1*15:01 positive	1.5 + 2.3	7.8	SI 3.8, p=0.03	77
Past IM HLA-DRB1*15:01 positive	1.2 + 2.5	7.4	Not calculated	81
Past IM HLA-DRB1*15:01 positive HLA-A*02:01 negative	1.5 + 4.2 + 1.8	8.7	Total RERI 8.7 (4.7-12.6)	33

*Reference group was HLA-DRB1*15:01 negative; OR, odds ratio; EBNA, Epstein-Barr virus nuclear antigen; IM, infectious mononucleosis; HLA, human leukocyte antigen; AP, attributable proportion due to interaction, RERI, relative excess risk due to interaction; SI, synergy index. The total 3-way interaction takes all two-way interactions and the three-way interaction into account.

## Sun exposure and vitamin D

The increasing MS occurrence with distance from the equator (86, 87) has been attributed to lower sun exposure and consequently, lower endogenous production of vitamin D (82, 83). Observational studies have found higher risk of MS with lower sun exposure and with lower vitamin D levels. Although distinguishing between the effects of sun exposure and vitamin D may be challenging in observational studies, independent benefits have been observed when both factors have been considered simultaneously (88, 89). Sun exposure may influence MS risk through both vitamin D and non-vitamin D pathways, both of which impact innate and adaptive immune functions.

Vitamin D is converted enzymatically in the liver to its major circulating form (25-hydroxyvitamin D) and then in the kidney to its active form (1,25-dihydroxyvitamin D). Active vitamin D acts as a transcription factor by binding to the vitamin D receptor (VDR) and modulating the expression of over 200 genes. Vitamin D influences the immune system in multiple ways, including enhancing innate immunity, modulating adaptive immunity, and regulating cytokine production. It increases the differentiation of hematopoietic stem cells into NK cells and may enhance their function (90, 91). It also increases the production of antimicrobial peptides involved in directly killing virus (92). Vitamin D decreases the proliferation and differentiation of T cells and their cytokine production, while promoting the development and function of regulatory T cells. Both *in vitro* and *in vivo* studies show that vitamin D results in increased production of the anti-inflammatory cytokine IL-10 (93). The effects of vitamin D on B lymphocyte function are less certain. Although *in vitro* studies have provided some evidence that vitamin D inhibits the T cell-stimulating capacity of B cells and enhances the activity of regulatory B cells, it is not supported by *in vivo* data (94).

Exposure to UVR also has effects on the immune system that are independent of vitamin D, including induction of interferons that inhibit viral replication and enhancement of innate immune responses (95). Sun exposure also results in suppression of cell-mediated immunity, independent of vitamin D. Exposure to sun induces regulatory T cells through antigen presentation of sun-damaged Langerhans cells in the lymph nodes, leading to antigen-specific immune tolerance. Exposure to sun also inhibits effector and memory T cell activation, and induces systemic changes in immune mediators, leading to suppression of the adaptive immune response (96, 97). A suppressive effect of UVR through pathways independent of vitamin D have also been confirmed in several experimental studies (98, 99).

In patients with MS, an inverse relationship has been observed between EBNA-1 antibody levels and serum vitamin D levels (100, 101), suggesting that vitamin D deficiency may impair the T cell-mediated response to EBV, leading to viral reactivation and augmented antibody production. Low sun exposure/vitamin D deficiency has also been found to act synergistically with high levels of EBNA-1 antibodies regarding risk of MS (102). There is some evidence that vitamin D levels could affect the interaction between EBV and MS predisposing genes. EBNA2, a transcriptional activator with importance for B cell transformation, binds to MS susceptibility loci in the host genome of infected B cells and may influence their expression (103). An overlap between EBNA2 and VDR binding sites has been observed and it has been suggested that EBNA2 and VDR compete for binding to these susceptibility loci of which the majority is related to immune responses (104). Considering that EBV and vitamin D may have antagonistic effects on B cell function, adequate vitamin D levels could thus have a protective effect against MS by blocking EBNA2 genomic occupancy.

The influence of vitamin D on MS risk has been suggested to be important during a time window. While low vitamin D levels during the first trimester resulted in increased risk of MS in the offspring (105), studies investigating the association between neonatal vitamin D status and risk of subsequently developing MS have been conflicting (106, 107). However, several studies have consistently found decreased risk of MS with increasing vitamin D levels in childhood and young adulthood. In a prospective, nested case-control study among US military personnel, the inverse association between vitamin D levels and risk of MS was particularly strong for vitamin D measured before age 20 years (108).

The latitude gradient in MS occurrence and the association between increased risk of MS and both IM history and vitamin D deficiency have led to the hypothesis that the season of IM could influence MS risk (109). If vitamin D dependent responses against primary EBV infection affects the risk of MS, it would be expected that IM during winter, when vitamin D levels are at their lowest, confers a stronger risk of subsequently developing MS. However, the increased risk of MS following IM does not seem to be dependent on the season of infection (110, 111). One explanation could be that vitamin D deficiency has more long-term effects on the immune system, possibly by reducing the expression of key genes involved in T cell development and function.

## Lung-irritating agents

There is strong epidemiological evidence for an association between smoking and other lung-irritating agents and risk of MS. Smoking increases the risk of MS in a dose-response manner (112, 113). A similar dose-response correlation has been observed between increased risk of MS and exposure to passive smoking, organic solvents, and air pollution, respectively (114–117). These lung-irritating agents also display a considerable interaction with MS-associated risk genes (118–121). Since it seems unlikely that MS risk genes would regulate smoking behavior or other exposures to lung-irritating agents, it is an argument for a causal role of pulmonary irritation in MS development.

Tobacco smoking and its oxidative agents results in airway inflammation and increased levels of inflammatory markers (122–124). Alveolar macrophages in the lungs increase in numbers with concurrent impairment of their function, contributing to the inflammatory process. The development and effector function of both innate and adaptive immune cells are affected by smoking, including NK cells, CD8+ and CD4+ T cells as well as memory B and T lymphocytes. The majority of studies in humans have found that smoking increases the number of CD8+ T cells and enhances their functional response while the frequency of CD4+ T cells are reduced (122–124). Smoking also induces changes in the distribution and function of memory B cells, with a higher prevalence of memory B cells in peripheral blood and in the lung, whereas regulatory B cell numbers decrease (125).

Smoking may act synergistically with high levels of EBNA-1 antibodies to increase the risk of MS ( (126–128), **Table 2**). Apart from inducing proinflammatory responses, smoking results in a relative immune deficiency (123). Smokers have higher EBNA-1 antibody levels than non-smokers both among patients with MS and healthy controls (128–130), and smoking has been associated with frequent reactivation of EBV whereby EBV antibodies against viral antigens become elevated. There is a dose-response relationship between smoking and both oral EBV load, anti-EBV IgG levels, and the frequency of EBV reactivation (131, 132). This is also supported by experimental evidence showing replication of EBV with expression of its lytic-phase genes following exposure to cigarette smoke (133). Smoking-associated DNA methylation and changes in gene expression in immune cell types have been identified and may contribute to EBV reactivation (134).

**Table 2:** Compilation of studies examining the additive interactions between Epstein-Barr virus and smoking in relation to the development of MS.

Risk factors	OR for each risk factor in the absence of the other/s	Combined OR	Additive interaction	Reference
High anti-EBNA Ever smoking	1.8 + 2.5	2.7	SI 0.7, p=0.6	77
Anti-EBNA>median Ever smoking	2.7 + 1.5	3.8	AP 0.2 (0.1-0.3)	128
Anti-EBNA>median Current smoking	2.7 + 1.6	4.4	AP 0.3 (0.2-0.4)	128
Anti-EBNA>75^th^ percentile Ever smoking	4.1 + 0.6	7.4	AP 0.4 (0.1-0.7)	126
Past IM Ever smoking	1.5 + 2.1	3.2	SI 1.4, p=0.6	77
Past IM Current smoking	1.8 + 1.6	3.0	AP 0.2 (0.004-0.4)	128

OR, odds ratio; EBNA, Epstein-Barr virus nuclear antigen; IM, infectious mononucleosis; AP, attributable proportion due to interaction; SI, synergy index.

Smoking-associated pulmonary inflammation could contribute to the activation of tissue-resident immune cells within the lung including potentially autoreactive effector and memory cells. In experimental studies, CNS-directed cells in the lung become activated by local stimulation, whereby they assume a migratory pattern and traffic to the CNS (135). Smoking also affects the immune system barrier function and may promote migration of autoreactive immune cells into the CNS.

While some aspects of childhood or adolescence seem to be critical regarding the impact of several environmental factors on MS risk, smoking increases the risk of MS regardless of age at exposure (113). Following smoking cessation, the detrimental effect abates within a decade, even in heavy smokers with a long duration of smoking (113).

## Obesity

Several observational studies have observed that obesity in adolescence and young adulthood confers an approximately doubled risk of MS, compared to normal weight individuals (136, 137). The association is supported by Mendelian randomization studies showing associations between genetic predisposition to obesity and risk of MS (138–140).

Obesity induces a chronic low-grade systemic inflammation due to increased numbers of adipose tissue macrophages that undergo a phenotypic switch with increased production and secretion of inflammatory mediators (141). Several aspects of the adaptive system are affected, including increased proliferation and activation of T cells and reduced numbers of regulatory T cells, which exacerbate adipose tissue inflammation and could promote the onset of autoimmune responses (142, 143). The inflammatory pathways activated by obesity also upregulate pro-inflammatory cytokines and cause a decrease in the blood-brain barrier integrity, allowing for easier immune penetration (144). Apart from increased proinflammatory responses, obesity results in a relative state of immunodeficiency which increases susceptibility to infections (145). Obesity has been associated with a reduced function of NK cells (146), which are critical during primary EBV infection. Obesity could also have impact on the immune response to EBV infection by contributing to dysbiosis (147).

Obesity at young age is associated with subsequent risk of MS (148, 149) while obesity at the time of MS diagnosis does not differ between cases and healthy controls (148). It has also been suggested that obesity during adolescence rather than during childhood, is of importance for adulthood-onset MS, suggesting that obesity at the time of primary EBV infection may be of importance (150). In two studies, adolescent BMI exceeding 25 kg/m^2^ interacted with both past IM and high EBNA-1 antibody levels regarding risk of MS (**Table 3**). Past IM in obese individuals carrying the HLA-DRB1*15:01 haplotype rendered a 22-fold increased risk of MS, compared with normal weight DRB1*15:01 negative individuals without history of IM. The interaction between BMI and EBNA-1 status became stronger with increasing EBNA-1 antibody levels (151, 152). The obesity-induced immunodeficiency may thus alter the immune response to EBV and may, particularly in DRB1*15:01 carriers, increase the risk of an autoreactive response directed at CNS-antigens.

**Table 3:** Compilation of studies examining the additive interactions between Epstein-Barr virus and overweight/obesity in relation to the development of MS.

Risk factors	OR for each risk factor in the absence of the other/s	Combined OR	Additive interaction	Reference
Anti-EBNA 50-75^th^ percentile* Adolescent BMI>25 kg/m^2^	2.1 + 1.5	2.7	AP 0.02 (-0.3-0.3)	151
Anti-EBNA 75-95^th^ percentile* Adolescent BMI>25 kg/m2	3.4 + 1.4	4.6	AP 0.3 (0.1-0.5)	151
Anti-EBNA >95^th^ percentile * Adolescent BMI>25 kg/m2	4.0 + 1.4	7.3	AP 0.4 (0.1-0.7)	151
Anti-EBNA>median HLA-DRB1*15:01 positive Adolescent BMI>25 kg/m2	2.5 + 2.8 + 1.6	13.5	Total RERI 8.6 (5.0-12.1)	151
Past IM Adolescent BMI>27 kg/m2	1.8 + 1.7	8.1	AP 0.7 (0.3-0.8) RERI 5.5 (1.7-12.8) SI 4.6 (2.0-10.2)	152
Past IM Adolescent BMI>27 kg/m2	1.8 + 1.6	7.0	0.7 (0.2-0.8) RERI 4.5 (1.1-11.2) SI 4.2 (1.7-10.2)	152
Past IM DRB1*1501 positive Adolescent BMI>25 kg/m2	2.0 + 3.4 + 1.4	22.2	Total RERI 15.4 (0.8-30.1)	151

*Reference group was EBNA-1 antibody levels below the median among controls; OR, odds ratio; EBNA, Epstein-Barr virus nuclear antigen; IM, infectious mononucleosis; BMI, body mass index; AP, attributable proportion due to interaction; RERI, relative excess risk due to interaction; SI, synergy index.

The total 3-way interaction takes all two-way interactions and the three-way interaction into account.

## Other potential risk factors

The gut microbiota plays a key role in regulating immune functions and has attracted major attention in MS (153). Intestinal microbiota has been shown to promote the triggering of CNS-reactive CD4+ T cells (154) and modulates astrocyte activity and neuroinflammation (155, 156). The microbiota composition in patients with MS has been reported to be distinct compared to healthy controls (157–159) and the transplantation of MS intestinal microbiota has been shown to induce or exacerbate experimental autoimmune encephalomyelitis (158, 159). Dysbiosis, which refers to an imbalance in the gut microbiota, has been associated with changes in the production and metabolism of fatty acids, which have immunomodulatory effects and modulate the differentiation of both innate and adaptive immune cells and their function (160). The level and composition of circulatory and tissue-resident fatty acids have been implicated in MS (160). Although the exact mechanisms by which the microbiome may affect the risk of MS remain uncertain, these findings highlight the importance of further research.

Polyunsaturated fatty acids (PUFAs) possess potent immunomodulatory effects (161). Several observational studies investigating the influence of PUFA dietary sources on the risk of MS have reported that a higher intake of fatty fish or supplementation with cod liver oil is associated with a reduced risk of developing the disease (162–165). However, the findings should be interpreted with caution due to potential sources of bias.

Although studies have not been consistent (165–167), sibship structure has been suggested to decrease the risk of MS by preventing delayed primary EBV infection (167).

Shift work, poor sleep, and psychological stress, all of which can negatively affect the immune response to infection, have also been associated with increased risk of MS (168–170).

## Preventive measures

Evidence strongly suggests that EBV is a prerequisite for MS and the most effective way to prevent the disease would be to prevent the primary EBV infection through vaccination. Various immunogens, including both lytic and latent proteins, have been explored for inclusion in prophylactic EBV vaccines (171), and several vaccines are currently undergoing clinical trials. Even if the primary infection is not prevented, a vaccine that prevents the excessive immune responses against primary EBV infection may be effective in reducing the incidence of MS. However, given that the onset of MS may occur decades after primary EBV infection, long-term follow-up studies will be necessary to evaluate the effectiveness of a vaccine in preventing MS, and it is important to identify alternative strategies to reduce the overall risk of the disease.

Several modifiable factors have consistently been associated with increased risk of MS that could be targeted through preventive measures. Based on the presented findings, one approach could be balanced information regarding the risks and benefits of sun exposure. The ultraviolet index can be useful for guiding sun exposure behavior in accordance with WHO guidelines to minimize the risk of skin cancer (172), but sensible sun exposure is essential for vitamin D synthesis and for capturing other vitamin D-independent benefits (95). Reducing the prevalence of smoking requires a multi-faceted approach that involves a combination of strategies, such as governmental regulations, prevention programs in schools, cessation programs for smokers, and increased access to smoking cessation resources. Parents should be informed regarding the importance of protecting their children from secondhand smoke. Childhood obesity is a serious and growing problem in developed countries. Preventive measures include encouraging healthy eating habits, promoting regular physical activity, limiting screen time, and encouraging healthy sleep habits. Healthcare professionals could be consulted when a child is at risk of becoming obese for guidance on appropriate nutrition and physical activity strategies. The health benefits of Mediterranean-style diet for obesity-related comorbidities are well-established (173). The diet is rich in fiber, vitamins, minerals, and antioxidants, and is based on consumption of high amounts of vegetables, fruits, nuts, fish and other seafood, and the use of olive oil (174). These guidelines align with those for general health benefits and may have the potential to lower the risk of MS.

## Conclusions

MS may arise from a complex interplay between latent EBV infection, genetic predisposition, and various environmental and lifestyle factors that negatively affect immune control of the infection. Increasing evidence from various research disciplines emphasizes the crucial involvement of EBV in the development of MS. EBV infection as a prerequisite for MS is further supported by evidence indicating that other established risk factors for MS act synergistically with EBV in the development of the disease. However, while numerous genetic variants and environmental factors have been identified that predispose to MS, their biological significance remains uncertain, and their effects are often small or modest. Certain environmental factors could have multiple biological effects that are significant in the pathogenesis, whereas other exposures may impact a small set of shared pathways, leading to comparable biological effects. Despite recent progress, gaining a comprehensive understanding of the various risk factors for MS onset remains a significant challenge. A multidisciplinary approach could provide further insight into the mechanisms of disease pathogenesis and provide important information for disease prevention that could be implemented.

## Author contributions

The author confirms being the sole contributor of this work and has approved it for publication.
